# Mechanoecology: biomechanical aspects of insect-plant interactions

**DOI:** 10.1007/s00359-024-01698-2

**Published:** 2024-03-14

**Authors:** Gianandrea Salerno, Manuela Rebora, Elena Gorb, Stanislav Gorb

**Affiliations:** 1 Dipartimento di Scienze Agrarie, Alimentari e Ambientali, University of Perugia, Borgo XX Giugno, Perugia, 06121 Italy; 2 Dipartimento di Chimica, Biologia e Biotecnologie, University of Perugia, Via Elce di Sotto 8, Perugia, 06121 Italy; 3 Department of Functional Morphology and Biomechanics, Zoological Institute, Kiel University, Am Botanischen Garten 9, 24098 Kiel, Germany

**Keywords:** Biomechanics, Adhesion, Friction, Surface, Adhesive pad, Ecology, Insects, Plants, Epicuticular wax

## Abstract

Plants and herbivorous insects as well as their natural enemies, such as predatory and parasitoid insects, are united by intricate relationships. During the long period of co-evolution with insects, plants developed a wide diversity of features to defence against herbivores and to attract pollinators and herbivores’ natural enemies. The chemical basis of insect-plant interactions is established and many examples are studied, where feeding and oviposition site selection of phytophagous insects are dependent on the plant’s secondary chemistry. However, often overlooked mechanical interactions between insects and plants can be rather crucial. In the context of mechanoecology, the evolution of plant surfaces and insect adhesive pads is an interesting example of competition between insect attachment systems and plant anti-attachment surfaces. The present review is focused on mechanical insect-plant interactions of some important pest species, such as the polyphagous Southern Green Stinkbug *Nezara viridula* and two frugivorous pest species, the polyphagous Mediterranean fruit fly *Ceratitis capitata* and the monophagous olive fruit fly *Bactrocera oleae*. Their ability to attach to plant surfaces characterised by different features such as waxes and trichomes is discussed. Some attention is paid also to Coccinellidae, whose interaction with plant leaf surfaces is substantial across all developmental stages in both phytophagous and predatory species that feed on herbivorous insects. Finally, the role of different kinds of anti-adhesive nanomaterials is discussed. They can reduce the attachment ability of insect pests to natural and artificial surfaces, potentially representing environmental friendly alternative methods to reduce insect pest impact in agriculture.

## Diversity of insect-plant interactions 

The majority of insect species are associated with plants: a large number of insect species are phytophagous and many non-phytophagous species are their predators or parasitoids. Such a relation requires from the insects an ability to climb up the plants, cut, grind, and penetrate plant tissues, lay eggs on plant surface, efficiently collect plant derivates, such as nectar, pollen grains, oils, etc. On the other hand, in such associations, plants often possess the ability to protect themselves mechanically by developing strong, sometimes mineralised, materials or by producing specialised surface coatings or features preventing insect attachment. Plants build mechanically or tribologically effective traps to capture and retain insects in order to digest them (carnivory plants) or use as pollination agents (flower traps).

In order to understand the principles of insect associations with plants, one has to consider not only chemical interactions between them, which have been relatively intensely studied in the past, but also to understand them from the mechanical point of view (Whitney and Federle 2013; Salerno et al. 2023a). Since the majority of the above mechanical interactions require contacts between insect body parts (mouthparts, legs, ovipositors, collecting devices, etc.) and plant organs (roots, stems, leaves, flowers, seeds, etc.), the contact problem dealing with an interaction should be considered (Gorb and Gorb 2009a). These contacting bodies may differ in their shapes, surface profiles as well as physico-chemical properties. Furthermore, insect surfaces are often covered by specialised adhesive or lubricating fluids, whereas plant surfaces may bear particulate materials (epicuticular wax projections, pollen grains) or fluids (resins, water-based saps, oils, etc.). This means that the real situation in contact is usually much more complex than a classical solid-solid contact without any third bodies (fluids, particles, etc.) in between.

Insects are engaged in various relationships with plants, including antagonism, commensalism, and mutualism, all of which significantly impact production in agriculture, horticulture, and forestry (Schoonhoven et al. 2005). Three key interactions — pollination, phytophagy, and plant carnivory — result from mutualistic or antagonistic coevolution between insects and plants.

Pollination represents one of the most important ecosystem services performed by insects (weighty also for human beings). It involves intricate cycles of pollen detachment and attachment, as it travels from the male to the receptive female part of different plants. In some plants, specialized kettle trap flowers have been developed in order to temporarily capture insect pollinators using a pitfall mechanism (Vogel and Martens 2000; Oelschlägel et al. 2009). The adhesive properties of pollen on plant and pollinator surfaces are crucial for understanding pollination processes (Ito and Gorb 2019).

Phytophagous insects equipped with chewing or piercing-sucking mouthparts can harm plants in diverse ways by consuming any part of a plant above or below the soil. These insects feed on primary metabolites (carbohydrates, lipids, and proteins) needed for their growth, while plants defend themselves through the production of secondary metabolites (alkaloids, terpenoids, and phenolic substances) and mechanical barriers that impede insect attachment, feeding, and oviposition.

Carnivorous plants, comprising approximately 810 vascular species, capture and digest animal prey, mainly insects, to absorb thus derived nutrients. Plant carnivory arises from complex adaptations to nutrient-poor habitats. Recent research has shed light on many aspects of these adaptations, but some aspects still remain unknown. Insects are lured by coloration and/or nectar, subsequently becoming permanently trapped and finely digested by trapping organs, such as lobster-pot traps, pitfall traps, flypaper traps, and pitchers, using trichomes (plant hairs), sticky mucilage or 3D epicuticular waxes (Jeffree 1986).

Given the complexity of insect-plant interactions, this review focuses on the biomechanical perspective of insect adaptations to attachment on plants and plant adaptations to prevent this. Numerous experimental studies including those by the authors of this review have been conducted in the last 10–15 years and a substantial level of understanding of various ecological questions related to insect attachment on plants has been reached. It is impossible to cover all abovementioned insect-plant interactions from the biomechanical perspective in one review. That is why we focus here on the insect adaptations to attachment on plants and plant anti-adhesive properties.

## Insect adaptations for attachment on plants and plant adaptations preventing insect attachment

Mechanisms of insect attachment depend on the particular surface profile of the plant substrate. Usually, insects use their sharp-pointed claws to grip onto the mesoscale surface asperities. However, the claw interlocking with surface irregularities is then successful, when the claw tip diameter is smaller than the characteristic dimensions of surface roughness (Dai et al. 2002). Insects possess two types of specialised structures called adhesive pads for building tight contact and in turn generating strong adhesion on smooth and micro-rough surfaces as well. These, so called smooth pads and setose (or hairy) pads, appeared several times independently in insect evolution (Beutel and Gorb 2001). The considerable softness of smooth pads’ material and the strong flexibility of fine hair-like cuticular structures (often called tenent setae) of the hairy pads cause an enhancement of contact area on different substrate textures (Gorb 2001). Interestingly, these attachment devices are not restricted to one particular site of the insect leg, but may be associated with various leg structures, such as claws, parts of the pretarsus, ventral surfaces of tarsomeres, or tibio-tarsal joint. Phylogenetic reconstructions of insects have revealed an independent origin of these structures in different recent insect groups (Beutel and Gorb 2001; Büscher and Gorb 2021).

Adhesive pads generate a secretory fluid into the contact with the plant substrate. This fluid consists of non-volatile, lipid-like compounds that have been previously detected in footprints by various histological techniques and methods of analytical chemistry (Ishii 1987; Kosaki and Yamaoka 1996; Eisner and Aneshansley 2000; Vötsch et al. 2002; Geiselhardt et al. 2009). Using high-resolution microscopy techniques, it was demonstrated that some insects produce fluids that represent a kind of micro-emulsion composed of two not mixable groups of substances: the water-soluble and lipid-soluble ones (Gorb 2001; Vötsch et al. 2002; Federle et al. 2002).

In general, pad adhesion relies on several basic physical forces. In an adhesion experiment on the bug *Rhodnius prolixus* Stål (Hemiptera: Reduviidae), Edwards and Tarkanian (1970) detected strong adhesion reduction in animals, which tarsi were treated with organic solvents. Stork’s (1980a) experiments with leaf beetles led him conclude that cohesive forces, surface tension, and molecular adhesion may contribute to the mechanism of insect attachment mediated by the pad fluid. Langer et al. (2004) applied atomic force microscopy in order to resolve forces on the tips of individual tenent setae in the fly *Calliphora vicina* Robineau-Desvoidy (Diptera: Calliphoridae) and clearly demonstrated that adhesion strongly depends on the presence of the pad fluid surrounded by the air. This is a strong evidence that insects use attractive capillary interactions mediated by the pad secretion between individual hair tips and the substrate.

The plant cuticle represents the interface between the organism and its environment. The cuticle itself and related structures might be considered as a functional organ, which properties reflect numerous influences of particular environment. Such environmental factors affect structural and chemical features of the cuticle. In the course of co-evolution between insects and flowering plants, the latter have evolved both surface chemistry and structure enabling proper attachment and locomotion of pollinating insects, whereas in the case of an antagonistic co-evolution, plants developed surfaces specialised in the reduction of insect attachment ability. The latter kind of interactions resulted for example in the development of surface-related defence strategies against herbivore insects and nectar robbers. In some special cases, it prevents insect escape from highly specialised traps of carnivorous plants.

In this review, we report shortly on the literature data and in main own recent experimental results on cuticular plant adaptations associated with insect–plant interactions. We aimed to demonstrate how plant surfaces can affect insect attachment abilities. In particular, the significance of 3D epicuticular waxes and trichomes is demonstrated in their role in insect attachment enhancement or insect attachment prevention. The structural and mechanical backgrounds of these interactions are discussed.

## Attachment organs in phytophagous insects

Phytophagous insects are polyphagous, oligophagous, and monophagous, reflecting their varying needs for primary metabolites related to nutrition, reproduction, habitat, and microclimate (Gullan and Cranston 2014). Our focus on insect-plant interactions concentrated on Hemiptera and Diptera species, which typically show smooth and hairy pads, respectively, and evolved a great variety of adhesive pad designs in relation to the necessity to attach on vegetation with various morphological features (Beutel and Gorb 2001). Special attention was given to the Southern Green Stinkbug *Nezara viridula* L. (Hemiptera: Pentatomidae), a polyphagous phytophagous insect, and two frugivorous species, the polyphagous Mediterranean fruit fly *Ceratitis capitata* Wiedemannand and the monophagous *Bactrocera oleae* Rossi (both Diptera: Tephritidae). *N. viridula* is a widespread pest that attacks over 30 crops globally and infests plants from at least 32 families (Panizzi et al. 2000). *C. capitata* and *B. oleae* are notorious pests, with *C. capitata* infesting over 220 fruit, nut, and vegetable species including various citrus species (White and Elson-Harris 1992; Liquido et al. 1998; CABI 2018) and *B. oleae* larvae being monophagous on olive fruit in the genus *Olea* including *O. europaea* L. On both cultivated and wild olives, *B. oleae* females lay eggs in ripening and ripe fruits that leads to significant losses in oil value reaching up to 80%.

In the Coleoptera order, specifically in Coccinellidae, the interaction with plant leaf surfaces is substantial across all developmental stages in both phytophagous and predatory species that feed on herbivorous insects. Our investigation focused on the attachment ability of all life stages of the phytophagous melon ladybird beetle *Chnootriba elaterii* (Rossi) (Coleoptera: Coccinellidae) to its host plants throughout its life stages. This oligophagous multivoltine species is a significant pest of Cucurbitaceae plants in Southern Europe as well as in the Near East, Middle East, and North Africa (Liotta 1964; Akandeh and Shishehbor 2011). The family Cucurbitaceae encompassing approximately 800 species across 130 genera (Jeffrey 2005) stand out as one of the economically vital plant families with many species characterized by the presence of trichomes on their leaves (Ali and Al-Hemaid 2011).

**Fig. 1:**
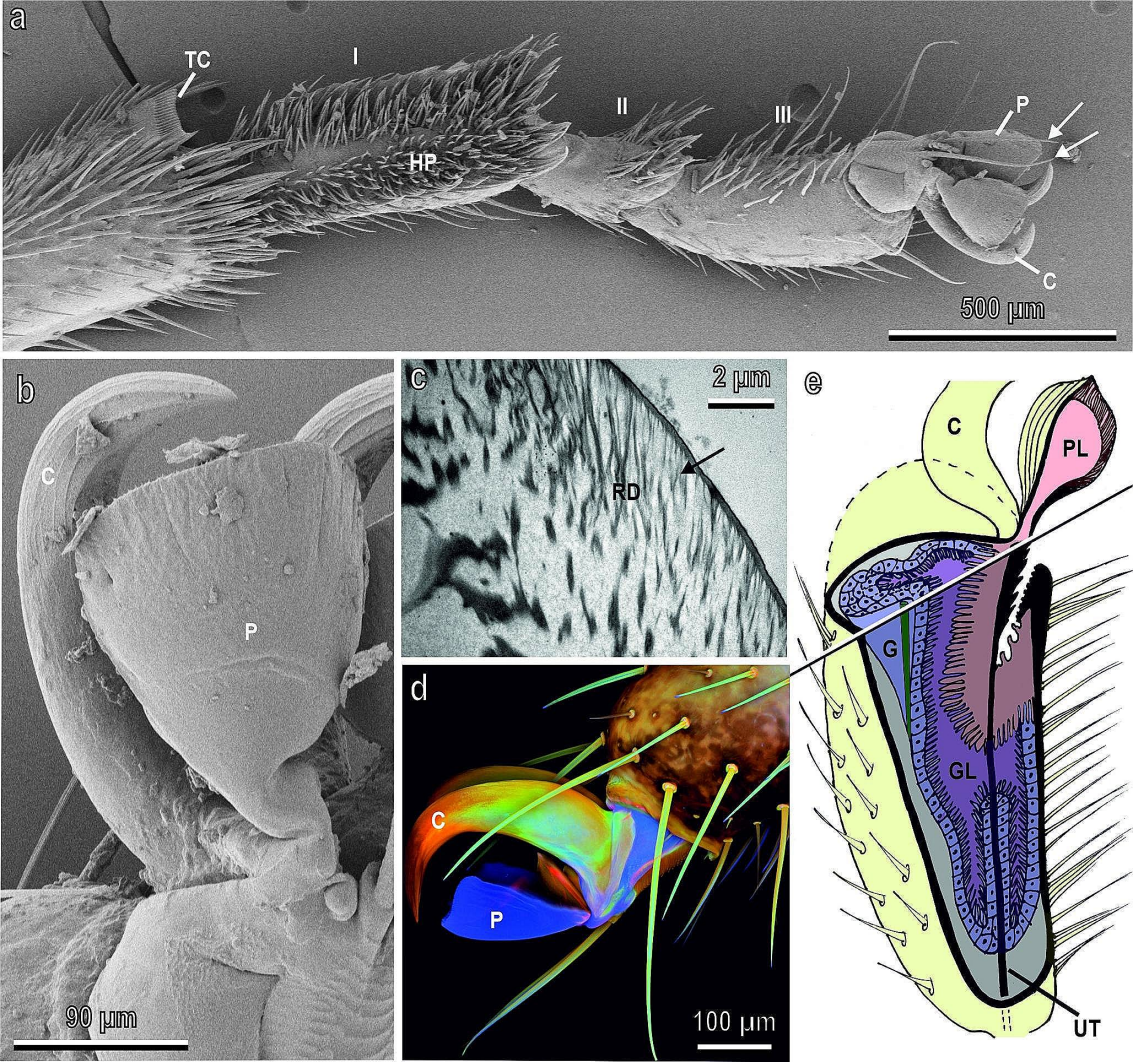
Tarsus of *Nezara viridula* in cryo-scanning electron microscopy (**a**, **b)**, transmission electron microscopy (**c)**, confocal laser scanning microscopy (**d)** and schematic reconstruction of the tarsal gland of *Coreus marginatus* (**e). a,** Ventro-lateral view of the three tarsal segments (I–III) and the pretarsus with pulvilli (P) and claws (C). Note the hairy pad (HP) on the basitarsus and the two paraempodia (arrows). TC, tibial comb complex used for antennal grooming. **b,** Ventral view of the pretarsus showing the smooth ventral surface of pulvilli (P) and the two curved claws (C). **c,** Detail of the ventral cuticle of the pulvillus in its distal portion showing the cuticular rods (RD) branching into thinner fibres towards the pad surface (arrow) and oriented at an angle of 40° to the surface plane. **d,** Lateral view of the pretarsus showing pulvilli (P) and claws (C). Differences in the autofluorescence composition of the exoskeleton structures reveal different chemical composition of the cuticle. Red colour indicates chitinous and strongly sclerotised exoskeleton structures, green colour indicates chitinous and non- or weakly-sclerotised exoskeleton structures, and blue colour indicates exoskeleton structures with large proportions of the very elastic and soft protein resilin. **e,** Schematic reconstruction of the tarsal gland. The third tarsomere is cut longitudinally and transversally. C, claws; G, gland; UT, unguitractor tendon. Note the gland lumen (GL) in connection with the pulvillus lumen (PL). **b**, **c**, and **d,** Modified from Rebora et al. (2018). **e** modified from Rebora et al. (2021)

The attachment devices of *N. viridula* are represented by a pair of pretarsal smooth pulvilli and a hairy pad on the ventral side of the basitarsus (Fig. 1a,b). An ultrastructural detailed investigation on the morphology of *N. viridula* attachment devices revealed no sexual dimorphism at different levels of the structural organization (Rebora et al. 2018). Pulvilli are sac-like structures formed by complex cuticular layers that vary in their morphology and resilin content (Fig. 1c,d). Footprints and contact areas of living resting individuals revealed that insect touches the substrate with the ventral surface of the distal portion of both paired pulvilli, with the distal portion of the basitarsal hairs and with the tips of the claws. In addition, the insect always touches the substrate with the distal portion of the two paraempodia (mechanosensory setae) to monitor the contact with the surface (Fig. 1a). The dorsal side of each pulvillus consists of sclerotised chitinous material, while the ventral cuticle contains strong proportion of resilin in the distal area, which is in contact with the substrate, and shows a very thin epicuticle and a thick exocuticle with cuticular rods branching into thinner fibres towards the pad surface and oriented at an angle of 40° to the surface plane (Fig. 1c). A similar ultrastructure has been found in the cuticular layers situated on the ventral side of the setal endplate of the pulvilli of *C. capitata* (Salerno et al. 2020a), where the cuticle branches into finer fibers to form thin cuticular rods (sometimes grouped in bundles) clearly oriented at an angle of 40° to the surface plane. The same ultrastructural fiber-like features have been described also in smooth and hairy attachment pads of other insect species (Slifer 1950; Roth and Willis 1952; Kendall 1970; Henning 1974; Hasenfuss 1999; Gorb et al. 2000; Betz and Mumm 2001; Federle et al. 2001; Perez-Goodwyn et al. 2006; Clemente and Federle 2008; Eimüller et al. 2008; Scholz et al. 2008; Schmitt and Betz 2017). In combination with the flexibility of resilin, which is present in large amount on the ventral side of smooth pads (Rebora et al. 2018) and also in the setal tips of the adhesive tarsal setae of beetles (Peisker et al. 2013; Gorb and Filippov 2014), the rods of the exocuticle could help the setal endplate to deform and replicate the surface profile, increasing by this the pad adaptability to rough surfaces (Gorb et al. 2000; Eimüller et al. 2008; Scholz et al. 2008).

The setae of the basitarsal hairy pad of *N. viridula* do not show any endplate but are pointed and socketed exhibiting a pronounced longitudinal and transverse gradient in the resilin content (Rebora et al. 2018). Traction force experiments on *N. viridula* with ablated pulvilli, shaved hairs and cut-off claws (Salerno et al. 2018a) revealed a great involvement of pulvilli in insect attachment on horizontal smooth and rough surfaces and during inverted climbing, which is often present in *N. viridula* especially during oviposition on the plant abaxial leaf side (Tood and Herzog 1980; Colazza and Bin 1995). The hairy pad seems to be important in attachment to horizontal hydrophobic substrates under water (Salerno et al. 2018a) and on vertical smooth roads as well (Voigt et al. 2019). The ability to adhere to hydrophobic substrates under water could be relevant for *N. viridula*, since many plants have a hydrophobic surface based on their lipophilic chemistry (Koch and Barthlott 2009) and may be often covered by a water film. The basitarsal hairy pad in *N. viridula* plays a multi-functional role since it is important not only as attachment device, but also in antennal grooming: the basitarsal hairy adhesive pad of one foreleg acts as a brush over the tibial comb complex of the opposite leg (Rebora et al. 2019).

*Nezara viridula* releases fluid onto the substrate and leaves traces of such fluid during each step (Ghazi-Bayat 1979; Ghazi-Bayat and Hasenfuss 1980; Rebora et al. 2018). A secreting epithelium is present in the adhesive pad in cockroaches (Betz et al. 2017), grasshoppers (Slifer 1950) and Mantophasmatodea (Eberhard et al. 2009) or at the base of the adhesive seta in beetles (Betz 2003), but the situation is different in the pulvilli of Hemiptera (Rebora et al. 2018, 2021). In *N. viridula*, similar to other Hemiptera such as *Coreus marginatus* (L.) (Hemiptera: Coreidae), the pulvillus cuticle has no epidermis or secretory epithelium underneath (Rebora et al. 2018). The same situation is in Hymenoptera, where arolia lack an epidermis and other structures (Federle et al. 2001; Jarau et al. 2005) but show a well developed tarsal gland with a lumen (the gland reservoir) separated from the hemolymph and in contact with the pulvillus lumen (Fig. 1e) (Rebora et al. 2021). The presence of a gland reservoir connected to the pulvillus lumen is coherent with a storage volume of adhesive fluid, which could have also a hydraulic function in order to increase the contact surface of pulvilli with the substrate (Rebora et al. 2021).

**Fig. 2:**
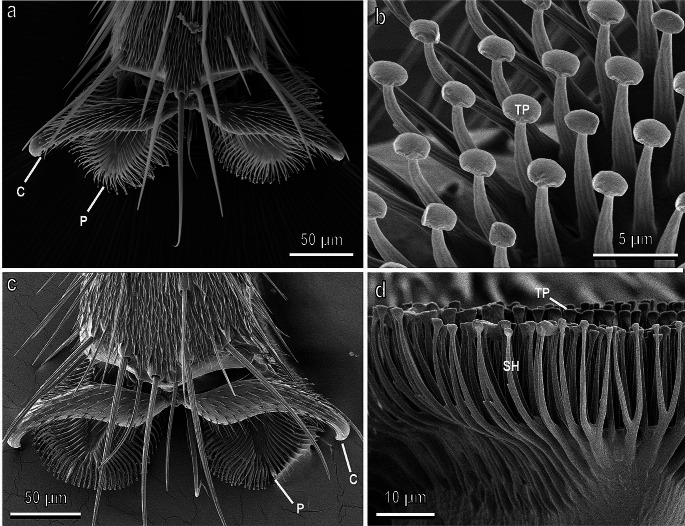
Pretarsal attachment devices of the female of *Ceratitis capitata* (**a**, **b)** and *Bactrocera oleae* (**c**, **d)** in cryo-scanning electron microscopy. **a,** Dorsal view of hairy pulvilli (P) and curved claws (C). **b,** Detail of the ventral view of a pulvillus showing the tenent setae with the terminal plate (TP). **c,** Dorsal view of hairy pulvilli (P) and curved claws (C). **d,** Detail of the tenent setae constituted of a setal shaft (SH) and a circular terminal plate (TP). **a** and **b,** Modified from Salerno et al. (2020a). **c** and **d, ** Modified from Rebora et al. (2020b)

Tephritidae flies possess two claws, two hairy pulvilli and a central bristle-like empodium (Fig. 2a,c). Such structure is similar to that described in other brachyceran families, such as Calliphoridae (Bauchhenß and Renner 1977; Bauchhenß 1979; Walker et al. 1985; Gorb 1998; Niederegger et al. 2002; Gorb et al. 2012) and Syrphidae (Gorb 1998; Gorb et al. 2001). As in Calliphoridae and Syrphidae (Gorb 1998), pulvilli are covered with hundreds of capitate tenent setae (Fig. 2b,d), which can be categorized into two types (according to different shapes of terminal plates) in the proximal and distal portions of the pulvillus. In both *C. capitata* and *B. oleae*, the female pulvilli are wider than the male ones.

**Fig. 3:**
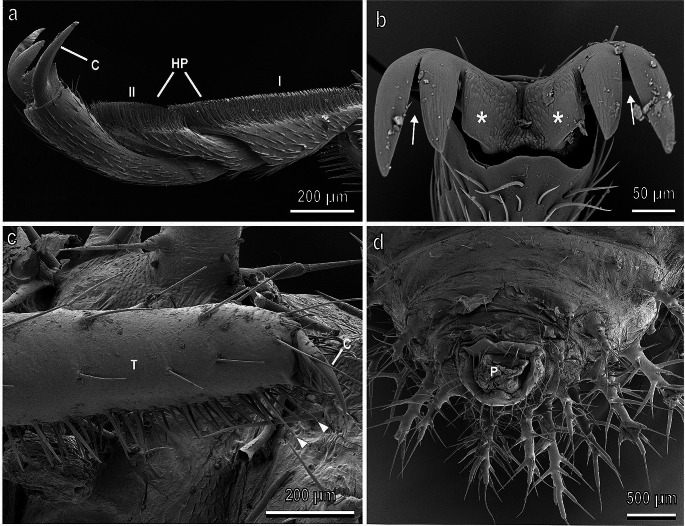
Tarsi of the female (**a**, **b)** and larva (**c**, **d)** of *Chnootriba elaterii* in cryo-scanning electron microscopy. **a,** Hairy pads (HP) covered with numerous tenent setae located on the ventral side of the first (I) and second (II) tarsal segments. C, claws. **b,** Frontal view of the bifid claws with a basal tooth (asterisk). Note the deep clefts (arrows). **c,** Single pretarsal claw (C) and tarsal tenent setae (arrowheads) located on the tarsus (T). **d,** Detail of the pygopodium (P) at the end of the abdomen. Modified from Saitta et al. (2022)

In Coccinellidae, tarsi are composed of four tarsal segments and the tarsal attachment organs consist of a pair of pretarsal claws and two hairy pads with numerous tenent setae (Fig. 3a,b) located on the ventral side of the first and second tarsal segments (Heepe et al. 2017; Gorb et al. 2019). Recent experimental data show that claws can be dentate (Fig. 3b) in relation with the aibility to interlock with flexible unbranched trichomes of different plants (Salerno et al. 2023b). Larval attachment devices are composed of a single pretarsal claw, tenent setae located on the tarsus (Fig. 3c) and a pygopodium or “anal organ” at the end of the abdomen (Fig. 3d).

## Insect attachment and plant cuticular folds

Microroughness asperity size of surfaces strongly reduces insect attachment ability in comparison with smooth surfaces or surfaces with a higher asperity size. Such effect has been shown in many insect species tested so far (Gorb 2001; Peressadko and Gorb 2004; Voigt et al. 2008; Wolff and Gorb 2012; Zhou et al. 2014; Zurek et al. 2017; Kovalev et al. 2018) and this was also the case in *N. viridula* (Salerno et al. 2017, 2018a) and *C.* *capitata* males and females (Salerno et al. 2020a) as well as in *Propylea quatuordecimpunctata* (L.) and *Harmonia axyridis* (Pallas) (both Coleoptera: Coccinellidae), both at the larval and adult stages (Salerno et al. 2022). This is due to the fact that tarsal adhesive pads can generate mainly contact on smooth substrate or smooth islands within rough substrates, whereas small surface irregularities reduce real contact area.

**Fig. 4:**
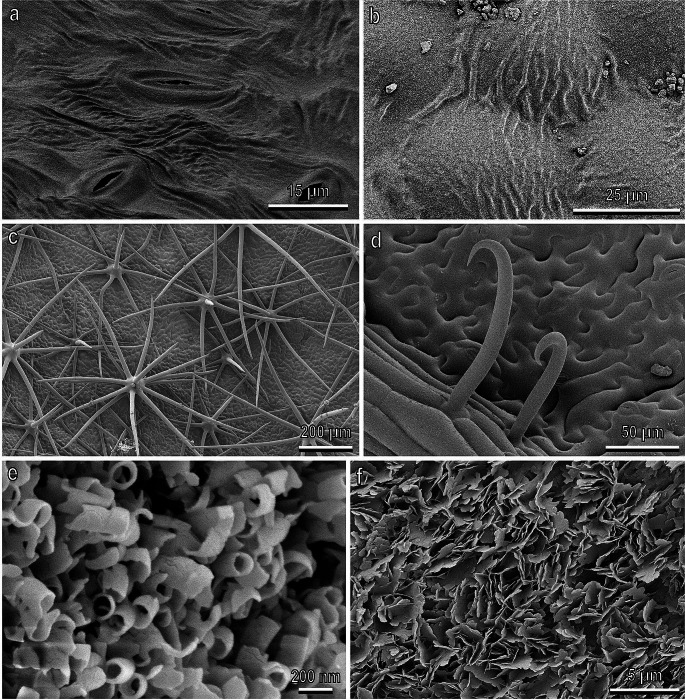
Leaf surface of *Prunus avium* (**a**), *Syringa vulgaris* (**b**), *Solanum melongena* (**c**), *Phaseolus vulgaris* (**d**) and fruit surface of *Prunus domestica* (**e**) and *Olea europaea* (**f**) in cryo-scanning electron microscopy. **a**, Abaxial leaf surface showing cuticular folds. **b**, Adaxial leaf surface showing cuticular folds running between neighboring cells. **c**, Adaxial leaf surface showing non-glandular branched trichomes. **d**, Adaxial leaf surface showing hooked non-glandular trichomes. **e**, Fruit surface characterized by a dense and regular 3D epicuticular wax coverage composed of numerous, very short, thin-walled tubules. **f**, Epicuticular wax coverage composed of platelets having irregular sinuate margins and protruding from the surface at different angles. **a**, Modified from Salerno et al. (2017). **b**), **c**, **d**, Modified from Salerno et al. (2018b). **e**, Modified from Salerno et al. (2020b). **f**, Modified from Rebora et al. (2020b)

Such effect of reduction in insect attachment ability due to microroughness is exploited by plant surfaces having cuticular folds (Fig. 4a,b). Based on a comparative SEM study of the functional surfaces in carnivorous plants and kettle trap flowers, the folds found in 11 species were regarded as structures preventing adhesion of insect pads due to contact area reduction caused by surface microroughness created by folds (Poppinga et al. 2010). In *N. viridula* walking on both leaf sides of *Syringa vulgaris* L. (Oleaceae), we observed a lower traction force, if compared to the leaf surface of other plant species (Salerno et al. 2018b). This reduction in attachment ability can be due to the numerous fine cuticular folds densely covering the adaxial and abaxial surfaces of this plant species (Salerno et al. 2018b). The effect of the cuticular folds has been studied in detail using traction experiments with the Colorado potato beetle *Leptinotarsa decemlineata* Say (Coleoptera: Chrysomelidae) walking on five plant surfaces with the folds of different magnitude (Prüm et al. 2012). These cuticular folds reduced strongly (especially the leaf surfaces with medium cuticular folds) the beetle adhesion in comparison to smooth plant surfaces without folds.

## Insect attachment and plant trichomes

Experiments examining the friction force of adult *N. viridula*, when attaching to various host plant species with smooth, hairy, and waxy surfaces revealed intriguing findings. Specifically, the abundant pubescence on *Solanum melongena* L. (Solanaceae) leaf surfaces, consisting of non-glandular stellate trichomes with vertical and accumbent side arms (Fig. 4c), was found not to decrease insect attachment. Surprisingly, these trichomes were observed to be utilized by insect claws to enhance attachment during pulling (Salerno et al. 2018b). Despite the well-established role of plant trichomes in serving as a defence mechanism against herbivores, certain insects on specific plants use trichomes as an additional “foothold” potentially facilitating their attachment to the plant, as noted by previous studies (Southwood 1986; Stork 1980b; Gorb and Gorb 2002; Voigt et al. 2007). Also in the experiments with *C. capitata*, rather high friction force was recorded on the fruit surface of *Prunus persica* (L.) Batsch (Rosaceae), which is characterized by a dense pubescence consisting of short and long, densely packed non-glandular trichomes (Salerno et al. 2020a).

Attachment tests conducted on the beetle *Phaedon cochleariae* F. (Coleoptera: Chrysomelidae) using various mutants of *Brassica* sp. (Brassicaceae) revealed that trichomes may serve as clinging sites for the beetle’s tarsal claws (Stork 1980b). In the case of the beetle *Chrysolina fastuosa* Scopoli (Coleoptera: Chrysomelidae), experiments assessing attachment to hairy plant substrates with trichomes of varying sizes demonstrated that the beetles attached well to all tested surfaces: the size and density of trichomes appeared to have no significant impact on attachment (Gorb and Gorb 2002). The omnivorous mirid bug *Dicyphus errans* Wolff (Hemiptera: Miridae), which resides on pubescent plants, exhibits both morphological adaptations, such as elongated curved claws, long and slender legs, and behavioral adaptations, including specialized locomotion, for navigating hairy plant substrates. Measurements of traction force on different hairy plants, including *S. melongena*, revealed a significant positive correlation between the force and both trichome length and diameter (Voigt et al. 2007).

The attachment ability of the oligophagous melon ladybird beetle *C. elaterii* at different developmental stages (adult, larva, eggs) to leaves of several Cucurbitaceae species (watermelon, melon, cucumber, zucchini, pumpkin, squirting cucumber, calabash and loofah) characterized by the presence of glandular and non-glandular trichomes having different length and density (Saitta et al. 2022) showed that glandular trichomes in Cucurbitaceae have no impeding effect on attachment of insects at both adult and larval stages, indicating an adaptation of *C. elaterii* to its host plants. However, non-glandular trichomes, especially when they are dense, short, and flexible, significantly diminish the attachment ability of both larvae and adults. In cases of dense but rigid trichomes, only the force exerted by larvae was reduced probably because larvae possess a single claw, in contrast to adults with paired bifid dentate claws. The investigation conducted by Salerno et al. (2023b) demonstrated that the specialized bifid dentate claws in adult *C. elaterii* represent an adaptation for interlocking with flexible unbranched trichomes found in some Cucurbitaceae, such as *Cucurbita moschata* Duchesne ex Poir. (Cucurbitaceae). In this study, the attachment ability of three Coleoptera species, *C. elaterii*, *H. axyridis* (both Coleoptera: Coccinellidae), and *C. herbacea* (Coleoptera: Chrysomelidae) having claws of different shapes was compared. It was found that plant trichomes can enhance insect attachment to plant surfaces compared with the smooth substrate (glass) by increasing interlocking effect of insect tarsal structures. This effect was directly related to the trichome stiffness of particular plant surface. To effectively grasp soft trichomes, insects evolved special claws-associated structures, such as the dentate claws observed in Coccinellidae (Fig. 3b) (Salerno et al. 2023b).

The ability of the hook-shaped trichomes of *Phaseolus vulgaris* L. (Fabaceae) (Fig. 4d) to entrap small insects such as leafhoppers, aphids, flies and bed bugs by impaling them is well-known (Gepp 1977; McKinney 1938; Pillemer and Tingey 1976, 1978; Szyndler et al. 2013; Rebora et al. 2020a). Our investigations on *N. viridula* characterised by a bigger size that were pulling on *P. vulgaris* leaf revealed that these trichomes are able to penetrate deeply inside the ventral surface of pulvilli and break at certain loading conditions (Salerno et al. 2018b). We observed a reduction in traction force when insects walked on glass after walking on *P. phaseolus* leaf due to the damage of their tarsal and pretarsal structures by the hooked trichomes.

## Insect attachment and plant waxes

The interaction between plants and their environment is mediated by a series of complex chemical and physical factors, among which epicuticular waxes (EWs) covering the surface of most plant organs have a fundamental functional role (Barthlott et al. 1998; Bargel et al. 2006). EWs are a complex mixture of cyclic (triterpenoids) or long chain aliphatic substances, such as primary and secondary alcohols, primary aldehydes, fatty acids and alkenes (Barthlott et al. 1998; Jetter et al. 2006), and constitute two-dimensional films/layers or three-dimensional micro- or nanoscale projections (Fig. 4e,f) covering the plant cuticle (Jeffree 1986). EWs represent the primary barrier against biotic and abiotic stress, exhibiting a multitude of functions such as being a barrier against water loss (Riederer and Schreiber 2001), offering protection against incident radiation by favoring light reflection (Barnes and Cardoso-Vilhena 1996), protection from surface contamination by dust particles (Barthlott and Neinhuis 1997; Fürstner et al. 2005) or from pathogenic microorganisms (Garcia et al. 1997). Plant surfaces with 3D epicuticular waxes have been shown to strongly reduce insect adhesion (Stork 1980b; Atkin and Hamilton 1982; Bodnaryk 1992; Brennan and Weinbaum 2001; Gaume et al. 2002, 2004; Chang et al. 2006; Gorb et al. 2008).

The very prominent and complex 3D epicuticular wax that covers both sides of the leaf surface of *Brassica oleracea* L. (Brassicaceae) induces a strong reduction in traction force of *N*. *viridula* (Salerno et al. 2018b). Likewise, a strong reduction in friction force was recorded in *C. capitata* on the fruit surface of *Prunus domestica* L. (Rosaceae) due to the dense and regular 3D epicuticular wax coverage composed of numerous, very short, thin-walled tubules oriented at different angles to the surface and making the fruit surface superhydrophobic (Fig. 4e) (Salerno et al. 2020a). Interestingly, the same reduction in friction force due to waxes recorded in the polyphagous *C. capitata* has been observed also in another tephritid species, the monophagous olive fruit fly *B. oleae*, which feeds exclusively on wild and cultivated olives typically characterised by the presence of waxes (Fig. 4f) (Rebora et al. 2020b). The experiments demonstrated that the attachment ability of the latter insect to the dewaxed olive fruit was higher than that to the wax-bearing one (Rebora et al. 2020b), thus revealing that waxy surfaces represent a challenge also for very specialised species, such as *B. oleae*. Moreover, it was shown that *B. oleae* shows a different ability to attach to the ripe olive surface of different cultivars of *O. europaea*. EWs appear in many different morphological forms (review in Bartlott et al. 1998), whose structure is strictly dependent on chemical composition (Bartlott et al. 1998). A considerable variability in the morphology of the olive epicuticular wax has been documented across different cultivars of *O. europaea* (Lanza and Di Serio 2015). It is worth noting that insect attachment relies not only on the presence of three-dimensional wax, but also on such wax characteristics as projection size, density of EW coverage, and the distribution of individual projections. This has been demonstrated in studies involving the ladybird beetle *Cryptolaemus montrouzieri* Mulsant (Coleoptera, Coccinellidae) walking on *Pisum sativum* L. (Fabaceae) plants with wild-type waxes and with the reduced wax coverage caused by particular mutation (Gorb et al. 2008). Similar findings were observed in another ladybird beetle *Coccinella septempunctata* L. (Coleoptera, Coccinellidae) on bio-inspired wax surfaces formed by four alkanes of varying chain length (Gorb et al. 2014). In light of these results, we propose that different patterns of EWs on the fruits of various *O. europaea* cultivars generate varying microroughness levels. This, in turn, results in different degrees of wettability and varying real contact area between the terminal contact elements of the fly setae and plant EW projections. As a consequence, the attachment ability of *B. oleae* females to the olive surface is likely to be influenced by these differences in EW morphology among cultivars. Further studies are needed to clarify these aspects in this important pest species.

Several explanations have been put forward to elucidate the phenomenon of insect attachment reduction caused by plant waxes (Gorb and Gorb 2002, 2013):

### Roughness hypothesis

This hypothesis assumes that the reduction in adhesion is attributed to the micro-roughness of the epicuticular wax coverage composed by wax projections with a nominal asperity size ranging usually between 0.3 and 1.0 µm in most wax-covered plant surfaces.

### Contamination hypothesis

According to this hypothesis, detached wax crystals contaminate insect attachment pads and act as a separation layer between the latter and the plant surface.

### Fluid-absorption hypothesis

This suggests that the absorption of pad fluid is facilitated by the structured wax coverage on the plant surface.

### Wax-dissolving hypothesis

This hypothesis proposes that the dissolution of wax crystals occurs due to pad secretion, leading to substrate slipperiness and hydroplaning.

Various authors tested the first three hypothesis (Gorb and Gorb 2006; Voigt et al. 2008; Scholz et al. 2010; Gorb et al. 2010, 2014, 2017, 2019; Borodich et al. 2010; England et al. 2016), thus providing evidences of their validity.

## Attachment of different larval instars: scaling effects

Owing to their smaller surface-to-volume ratio, larger animals are typically expected to attach less well (with lower safety factor) to surfaces and for this reason they evolved overproportionally large pads or adaptations increasing attachment efficiency, such as splitting up the contact into finer subcontacts (Arzt et al. 2003; Labonte and Federle 2015). Study on the attachment ability of different species of Syrphidae flies with various body dimensions revealed lower safety factor in the species having higher weight and size and the highest safety factor in the smallest species (Gorb et al. 2001). This tendency is widespread in insects and has been demonstrated also in other groups. For example in *C. marginatus*, due to different scaling of body mass and area of attachment organs, smaller insects (younger nymphal stages) attach relatively more strongly than adults (Gorb and Gorb 2004). Likewise, the analysis of the attachment ability of *N. viridula* during its nymphal development showed that the safety factor of the youngest and smallest tested nymphal instar (maximum recorded value: 192 in N2) was dramatically higher than that of all the other instars (minimum recorded value: 7 in N4) (Salerno et al. 2020b). In order to compensate for such a decrease of weight-specific attachment, large animals can develop (1) wider attachment devices or (2) adaptations increasing attachment efficiency (adhesion or friction per unit contact area) (Gorb and Gorb 2004; Labonte and Federle 2015). As expected, *N. viridula* of each instar (from N2 to adult) has progressively increased both the length of the pulvilli and the number of the basitarsal adhesive setae. The increase of pulvilli size has been observed also in *C. marginatus* (Gorb and Gorb 2004), but in this species the frictional properties of the pulvilli do not change during ontogenesis. In *N. viridula*, the safety factor was similar in N3, N4, N5 and in the adult probably due to the increased efficiency of pulvilli during development (Salerno et al. 2020b). The increase in pad efficiency with body size observed in *N. viridula* could be relevant for understanding how do larger animals respond to the loss of weight-specific adhesion and could be coherent with the need of this important phytophagous pest insect in keeping a strong attachment to the host plant during all its nymphal instars.

## Egg attachment to the plant surfaces

**Fig. 5:**
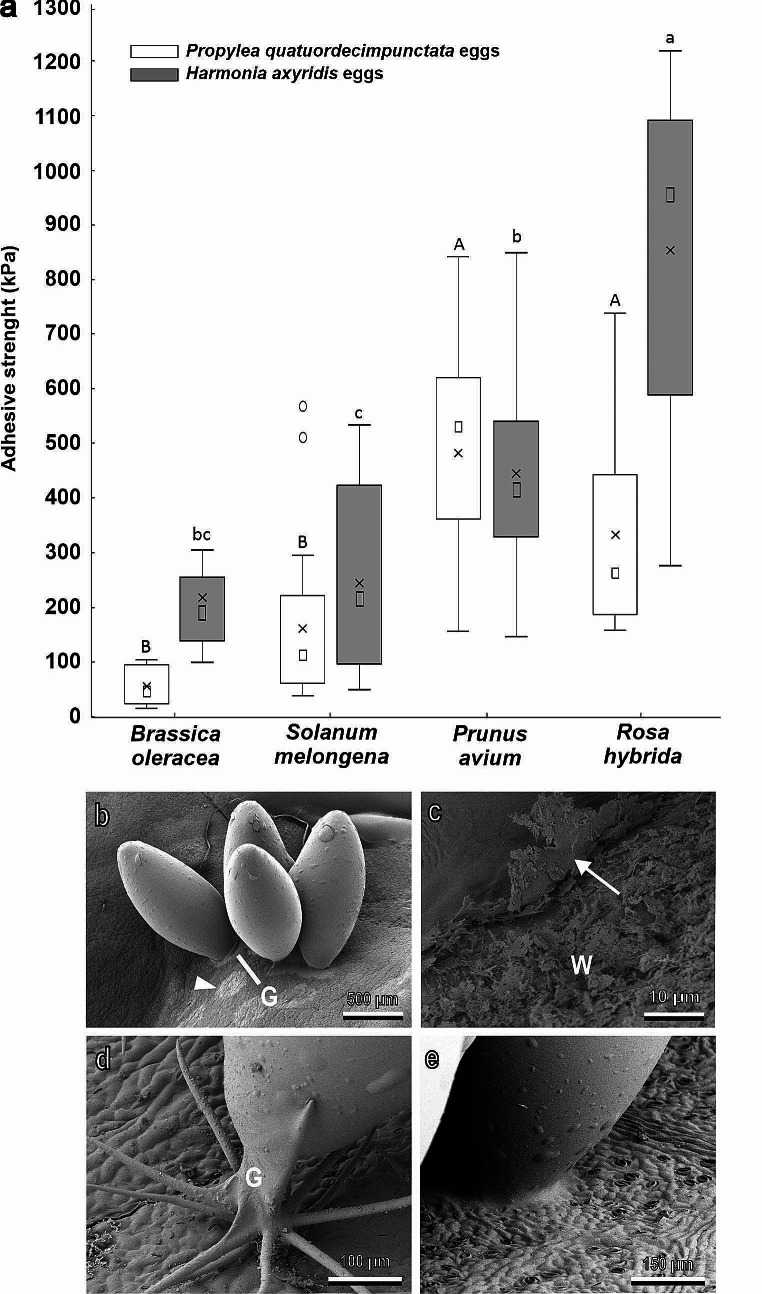
Egg adhesion (**a**) of two ladybird species to plant leaves characterized by different morphological features visible in cryo-scanning electron microscopy (**b-e**). **a**, Note that the higher reduction in adhesive strength is recorded on surfaces bearing waxes (*Brassica oleracea*) or trichomes (*Solanum melongena*). **b, c**, Egg interaction with the wax projections of *B. oleracea*. Note that the glue (G) adheres to the waxes (W), but these last detach easily from the leaf surface (arrow) leaving egg glue prints (arrow head) on the leaf. **d**, Egg interaction with the big stellate trichomes of *S. melongena*, which did not allow the egg glue (G) to reach the leaf surface. **e**, Egg interaction with the leaf of *Rosa hybrida*. The egg glue adheres well to the leaf surface, thus replicating the leaf topography. Modified from Salerno et al. (2022)

A firm attachment of the egg to the plant surface is fundamental for larval survival in both phytophagous insect species and their predators. For this reason, insects evolved adhesive secretions consisting largely of proteins (Li et al. 2008; Büscher et al. 2020). The mechanical interaction between insect egg glue and plant physical barriers represents an interesting aspect of insect adhesion. Coccinellidae egg glue has a high protein content (Li et al. 2008) forming both a very thin layer on the egg chorion and a basal disc able to keep the egg anchored to the surface (Fig. 5b-e). In this context, we studied the attachment ability of eggs of different species of Coccinellidae (both phytophagous and predatory species) to surfaces of different plant species. Leaf trichomes can reduce coccinellid egg adhesion compared to smooth leaf surfaces, as demonstrated for the eggs of *H. axyridis* and *P. quatuordecimpunctata* laid on the stellate trichomes of *S. melongena* (Fig. 5a,d) (Salerno et al. 2022). The cryo-SEM analysis of the interface between the base of *C. elaterii* eggs and the abaxial side of leaves of different Cucurbitaceae shows that in plant species with dense pubescence, such as *Lagenaria siceraria* (Molina) Standl. (Cucurbitaceae*)*, the glue cannot reach the leaf surface and the trichomes keep the egg away from the leaf surface, while in plants with a low trichomes abundance, such as *Cucumber sativus* L. (Cucurbitaceae), the glue readily spreads over the leaf surface (Saitta et al. 2022). In the presence of a thick layer of 3D waxes, such as in *B. oleracea*, the ladybird egg glue can adhere to the wax coverage, but the wax projections detach very easily from the leaf surface, thus causing the detachment of the eggs (Fig. b,c) (Salerno et al. 2022). The egg glue produced by *P. quatuordecimpunctata* and *H. axyridis* is similar to the egg glue observed in the asparagus beetle *Crioceris asparagi* (L.) (Coleoptera: Chrysomelidae) adhering to the waxy leaves of its host plant (Voigt and Gorb 2010). Specifically, the egg glue has the capability to wet the wax projections and integrate them into the adhesive matrix. These properties are similar to those found in the egg glue of *Cydia pomonella* L. (Lepidoptera: Tortricidae) able to wet the hydrophobic apple fruit surface (Al Bitar et al. 2014). The egg detachment happens easily due to the fracture of wax projections or their separation from the plant epidermis, as was shown by our cryo-SEM observations and force experiments (Fig. 5a,c), thus making the wax an effective protective structure against insect adhesion not only at the adult and larval stages, but also at the egg stage (Salerno et al. 2022).

## Potential implications for pest control

**Fig. 6:**
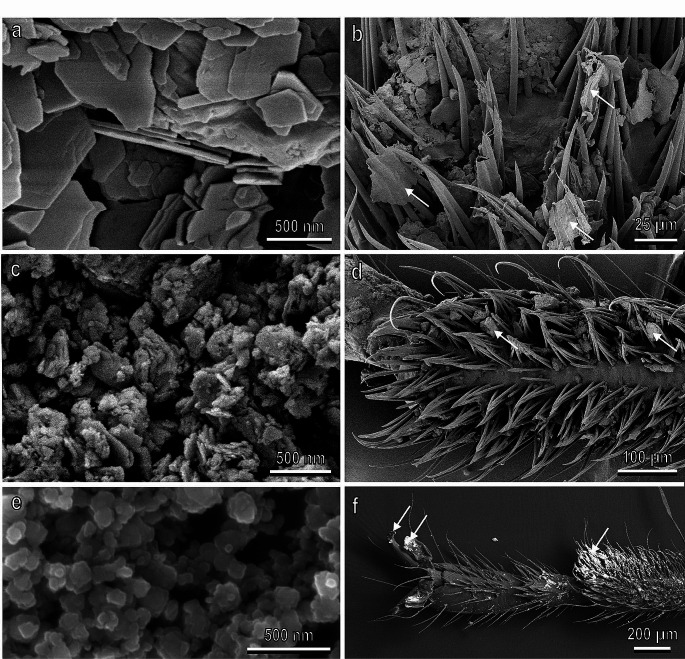
Anti-adhesive nanoparticles (**a, c, e**) and tarsal attachment devices of *Nezara viridula* after walking on treated hydrophilic glass (**b, d,f**) in cryo-scanning electron microscopy. **a**, Hexagonal- or pseudo-hexagonal-shaped, horizontally placed plates of kaolin particle film. **b**, Kaolin powder plates (arrows) accumulated among the adhesive setae of the basitarsal hairy pad. **c**, Plates of zeolite with variable shapes and dimensions forming groups oriented at different angles to the surface. **d**, Zeolite particles (arrows) strictly adhering to the adhesive setae. **e**, Biogenic zinc oxide nanoparticles (ZnO-NPs). **f**, Ventral side of the tarsal attachment devices of *N. viridula* just after insects walked on glass treated with biogenic ZnO-NPs (visualized with SEM, backscattered electrons). Note the white zinc oxide nanoparticles (arrows)

Owing to the significant repercussions of insect infestation on human well-being and economy, there has been a notable surge in human-initiated efforts to counter insects. Present approaches to control insect infestation frequently involve the use of harmful chemicals, such as insecticides and other repellents. Given the environmental hazards associated with these toxic substances, there is a growing need for alternative, eco-friendly strategies (Féat et al. 2019). Natural systems offer established biological principles that go beyond dependence on chemical defences, incorporating physical defence mechanisms, often achieved through surface structuring. From the studies of insect adhesion on plants, it is possible to assume that the most effective anti-adhesive systems are based on 3D wax coverage. That is why cost effective environment-friendly nanoparticle coatings might be a promising way for mechanical plant protection. Indeed, many recent technological examples derive their innovative function from simple observation and replication of solutions typical for living systems (Koch et al. 2009). In this context, the development of environmental friendly anti-adhesive coatings can represent an alternative method to reduce insect pest impact in agriculture. In our investigations, we demonstrated that different kinds of nanomaterials (Fig. 6) can reduce the attachment ability of different insect pests to natural and artificial surfaces (Salerno et al. 2020c, 2021; Rebora et al. 2023). In particular, the effect of kaolin particle film (Fig. 6a) (Glenn and Puterka 2005) on the reduction of insect attachment ability of *N. viridula* and *C. capitata*, characterised by different types of adhesive pads, was recently tested under controlled conditions (Salerno et al. 2020c). Data demonstrated that insect adhesion in both tested species is heavily affected by the fine-grained, nanoplate-like aluminosilicate mineral. The degree of reduction of insect adhesion on the treated substrates compared to the untreated ones differed depending on the kind of treated substrate, owing to its initial wettability and morphology (presence of trichomes). Insect adhesive pad contamination by the kaolin nanoflakes in both smooth and hairy pads (Fig. 6b), resulting in a significant decrease of the insect friction force not only on a treated surface, but also on a clean one after walking on a treated surface, was experimentally observed (Salerno et al. 2020c). Such mechanism of action of kaolin particle film in reducing insect adhesion is similar to that described in the contamination hypothesis explaining the effect of epicuticular plant waxes on reducing insect attachment (Gorb and Gorb 2002, 2006).

The ability to reduce insect attachment was tested also for other particle films, such as zeolite (Fig. 6c) and calcium carbonate (Salerno et al. 2021). The coatings were more uniform and compact in the cases of kaolin and zeolite compared to calcium carbonate particle film. Moreover, both aluminosilicate particles (kaolin and zeolite) can more readily adhere to *N. viridula* attachment devices, whereas calcium carbonate particles appeared less adherent to the insect adhesive pads. Only the application of kaolin reduced insect linear speed during locomotion.

Other nanoparticles, which are able to reduce insect attachment, are represented by biogenic and non-biogenic zinc oxide nanoparticles (ZnO-NPs) (Fig. 6e) (Rebora et al. 2023). The concentration of both biogenic (synthesized using a plant extract) (Buono et al. 2021) and non-biogenic ZnO-NPs, required to significantly reduce the attachment ability of *N. viridula* to glass surface, is around 12.5 mg L^− i^ and the reduction in attachment ability was very strong − ca. 70%, reaching 90% reduction of the initial force from a concentration of 50 mg L^− g^. SEM analyses revealed that these particles aggregate on the attachment devices of *N. viridula* including the pulvilli the hairy pad and claws (Fig. 6f), thus disrupting the attachment mechanism. These results are particularly interesting, because ZnO-NPs can have other beneficial effect on crops such as improving seed germination, plant vigor index, physiological and biochemical traits, and biomass production (Sun et al. 2020; Mishra et al. 2023; Punitha et al. 2023). Further studies in the laboratory and in the field can shed light on the potential involvement of these nanoparticles in insect oviposition and feeding deterrence or in reducing the insect survival rate and mating success, as reported for other nanoparticle films, such as kaolin (Puterka et al. 2005).

## Further perspectives

In spite of a great number of publications on the effects of plant cuticle micromorphology on insect adhesion, there is a number of interesting problems, which still remain unresolved. Further promising direction for the future research is the question about animal adaptations to overcome the slipperiness of specialised plant surfaces. There are numerous highly-specialised arthropod species, which are strongly adapted to the plants covered by 3D epicuticular wax. Usually, their adaptations are behavioural ones, but these specialist animals often possess contact elements of particular shape and dimensions on their attachment pads or/and generate specific adhesive fluid composition compared to those in generalist species.

Finally, a further exotic direction would be the use of plant waxes for generating templates for the nanofabrication in materials science and nanotechnology. This can lead to development of surface coatings for technical surfaces with various useful properties, but also to development of sprays preventing insect attachments to plant surfaces, which are lacking 3D epicuticular waxes in nature (Gorb et al. 2014).

## Data Availability

This article has no additional data.
